# The Effect of Low Temperature Storage on the Lipid Quality of Fish, Either Alone or Combined with Alternative Preservation Technologies

**DOI:** 10.3390/foods13071097

**Published:** 2024-04-03

**Authors:** María Dolores Suárez-Medina, María Isabel Sáez-Casado, Tomás Martínez-Moya, Miguel Ángel Rincón-Cervera

**Affiliations:** 1 Department of Biology and Geology, CEIMAR, University of Almería, 04120 Almería, Spain; dsuarez@ual.es (M.D.S.-M.); msc880@ual.es (M.I.S.-C.); tomas@ual.es (T.M.-M.); 2 Institute of Nutrition and Food Technology, University of Chile, Santiago 7830490, Chile; 3 Food Technology Division, University of Almería, 04120 Almería, Spain

**Keywords:** marine foods, low temperature storage, lipid quality, polyunsaturated fatty acids, antioxidants

## Abstract

Marine foods are highly perishable products due to their high content of polyunsaturated fatty acids, which can be readily oxidized to form peroxides and secondary oxidation products, thus conferring such foods undesirable organoleptic characteristics and generating harmful compounds that are detrimental to the health of consumers. The use of preservation methods that minimize lipid oxidation is required in the fishing and aquaculture industries. Low temperature storage (chilling or freezing) is one of the most commonly used preservation methods for fish and seafood, although it has been shown that the oxidation of the lipid fraction of such products is partially but not completely inhibited at low temperatures. The extent of lipid oxidation depends on the species and the storage temperature and time, among other factors. This paper reviews the effect of low temperature storage on the lipid quality of fish, either alone or in combination with other preservation techniques. The use of antioxidant additives, high hydrostatic pressure, irradiation, ozonation, ultrasounds, pulsed electric fields, and the design of novel packaging can help preserve chilled or frozen fish products, although further research is needed to develop more efficient fish preservation processes from an economic, nutritional, sensory, and sustainable standpoint.

## 1. Marine Foods: Sources of Healthy Fatty Acids

Marine foods are the major source of very-long chain polyunsaturated fatty acids from the n-3 family (n-3 VLC-PUFA), with eicosapentaenoic acid (EPA, 20:5 n-3) and docosahexaenoic acid (DHA, 22:6 n-3) being two of the most widely studied n-3 VLC-PUFA in terms of their beneficial effects on human health. They play relevant roles in cell signaling, structural integrity and fluidity of cell membranes, and regulation of blood pressure, and their cardioprotective effects have been widely evidenced [1]. EPA and DHA are precursors of eicosanoids (prostaglandins, thromboxanes, and leukotrienes) and docosanoids (resolvins, protectins, and maresins), respectively, which are anti-inflammatory mediators involved in many important biological processes in the human body [1,2]. EPA and DHA can be endogenously synthesized from their metabolic precursor, α-linolenic acid (ALA, 18:3 n-3), which is a dietary essential fatty acid. However, such conversion is hindered by the rate-limiting activity of the Δ6-desaturase, which is the enzyme responsible for converting ALA to stearidonic acid (SDA, 18:4 n-3) [3]. Thus, EPA and DHA are considered “conditionally essential” and their intake through the diet is advisable. The recommended daily intake (RDI) for EPA + DHA for a healthy adult ranges from 250 to 500 mg/day according to some international organizations such as the European Food Safety Authority (EFSA) and the International Society for the Study of Fatty Acids and Lipids (ISSFAL). Such RDI can be satisfied with the consumption of two or three portions of fish per week, considering that the saponifiable fraction of fish lipids is generally characterized by a high proportion of n-3 VLC-PUFA [4,5,6].

Due to their high degree of unsaturation, n-3 VLC-PUFA are very sensitive to oxidative degradation. Despite many other biochemical and microbiological processes taking place simultaneously, lipid oxidation is the most important factor for quality deterioration and shortening the shelf life of marine foods [7,8,9]. Therefore, the need to design strategies to avoid or reduce lipid oxidation during processing and storage of marine products is a key issue. Accordingly, various methods have been developed to minimize or delay these deteriorative changes and maintain the nutritional quality of marine foods. Low temperature storage (chilling or freezing) has been the most widely used method for preserving marine foods, and although it partially inhibits most of the processes leading to quality deterioration, it does not completely prevent lipid oxidation and unfavorable changes in lipid quality and sensory properties. Consequently, technologies such as high hydrostatic pressure, vacuum packaging, irradiation, and the use of natural preservatives, among others, have been combined with low temperature storage in an attempt to extend the shelf life of marine foods by minimizing lipid oxidation. The current review provides an overview of studies conducted to assess the influence of different preservation methods on the fatty acid composition and lipid quality of different edible fish species.

## 2. Lipid Hydrolysis and Oxidation Processes

The lipid fraction of marine foods is a rich source of highly unsaturated PUFA, making them more perishable than many other food products of animal origin. VLC-PUFA are highly susceptible to oxidation due to the presence of methylene bridges between double bonds in the hydrocarbon chain, which are easily abstracted by free radicals [10]. Thus, fish and seafood are highly sensitive to autolysis, oxidation, and hydrolysis processes, which are the major causes of a reduced shelf life, changes in color and flavor, and loss of nutritional value [9]. Oxidative damage is a major concern during fish storage as it contributes to quality degradation by altering flavor, texture, and color [11].

**Figure 1:**
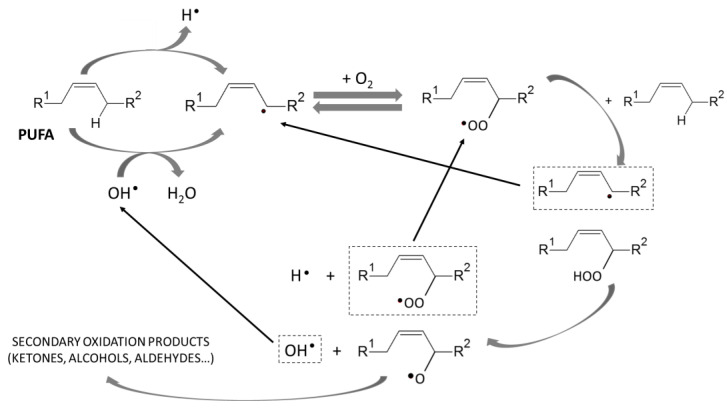
PUFA oxidative scheme to secondary oxidation products (adapted from [18]).

VLC-PUFA are mainly found as triacylglycerols (TAG) or phospholipids (PL) in fish tissues [5,6]. Both TAG and PL are hydrolyzed by the activity of lipases and phospholipases, increasing the proportion of free FA (FFA), which are susceptible to oxidation. The activity of lipases and phospholipases is species, tissue, and temperature dependent [12]. The oxidation of FFA involves the formation of free radicals, peroxides, and hydroperoxides, which are the primary products of lipid oxidation, and leads to the formation of secondary oxidation products (aldehydes, ketones, alcohols, and hydrocarbons, among others), many of which are responsible for the characteristic rancid off-flavors of degraded marine products [13] (Figure 1). Primary and secondary oxidation compounds have significant pro-oxidant activity and can cause damage to muscle proteins, leading to the formation of complexes with amino acids and protein denaturation, which in turn causes texture deterioration [14]. In addition, the potential production of unhealthy molecules associated with inflammatory, neurodegenerative, and cardiovascular diseases as well as cancer, atherosclerosis, or aging is another consequence derived from lipid oxidation [15].

The oxidative stability of fish tissues depends on an anti- and pro-oxidant balance. In living fish, there is a natural balance between the formation of free radicals and the endogenous antioxidant capacity of the animal itself, which is maintained by numerous intrinsic fish enzymatic systems such as superoxide dismutase (SOD), catalase (CAT), glutathione peroxidase (GPx), glutathione reductase (GR), and peroxiredoxins (Prxs), or non-enzymatic (tocopherols, biquinol, carotenoids, and ascorbate) and dietary antioxidant compounds. However, this protective function decreases significantly during post-mortem storage. FFA generated after hydrolysis of TAG and PL, together with other oxidation-promoting components (heme-proteins, metals, and pro-oxidant enzymes), play a key role to boost oxidative processes in marine foods [16]. Oxidative degradation is promoted by the exposure to high temperatures, light, oxygen, and handling conditions. Therefore, all processing steps of marine foods must be carefully controlled to prevent loss of sensory and nutritional quality.

N-3 VLC-PUFA available in PL are more susceptible to oxidation than those located in TAG because PL in cell membranes are more exposed to interactions with free radicals and closer to the catalytic sites of oxidative enzymes [13], whereas TAG are lipids with an energy reserve role and are mainly stored in the adipocytes [17].

## 3. Preservation Methods of Marine Foods

Traditional preservation methods for fish and seafood are based on storage at low temperatures, either chilling (0–4 °C) or freezing (<0 °C), as well as drying, smoking, salting, fermentation, and canning. Freezing is one of the most widely used preservation methods for fish due to its favorable quality/cost ratio and its low impact on the organoleptic and nutritional properties of the preserved products [19]. Several alternative methods, such as the use of natural preservatives, high hydrostatic pressures, ozonation, or irradiation, have been combined with cold preservation of fish in an attempt to minimize the oxidative degradation of fish lipids and therefore to maintain the quality of such products for a longer period of time [20]. Fifty-nine studies among those published in the last 25 years (between 1999 and 2023) that focused on assessing the quality of the fish lipid fraction during either low temperature storage alone or low temperature combined with other preservation techniques were reviewed in the current work.

### 3.1. Low Temperature Storage

Refrigerated storage (between 0 and 4 °C) is the most common method of preserving fresh fish. Icing is usually utilized as pre-treatment before a further refrigerated storage, being a crucial process in the fish industry during the fish slaughter and marketing chain, as it reduces the growth of microorganisms and avoids fish superficial dehydration [20]. Although frozen storage is one of the most widely used methods for fish quality preservation, lipase activity and FFA release cannot be completely avoided in frozen fish, especially at high or fluctuating temperatures, due to a disruption of the lysosomal membrane and a release of hydrolytic enzymes under these conditions [21]. Therefore, prolonged storage increases the possibility of lipid damage possibilities and favors the release of pro-oxidant compounds [18]. Storage temperature is the most important factor determining the processes of lipid hydrolysis and oxidation, as lipolytic rates are slowed at temperatures below −30 °C [22]. Freezing rate is also a key factor to maintain the quality of frozen foods. Ice crystals formed during rapid freezing are small and uniform, and are mainly located in the intracellular region, which protects the microstructure of the food [23].

Table 1 provides an overview of studies on the effect of storage temperature on the lipid quality and FA profile in different fish species. Common freezing temperatures for fish storage are between −18 and −25 °C, but several studies have shown that lipid quality can be negatively affected even at these temperatures after prolonged storage. A noticeable increase in FFA, peroxides (primary lipid oxidation products), and thiobarbituric acid reactive substances or TBARS (secondary lipid oxidation products) was noticed in Spanish mackerel (*Scomberomorus commerson*) and white cheek shark (*Carcharhinus dussumieri*) after 6 months of storage at −18 °C [24]. Significantly higher values of peroxide values (PV) (from 0.02 to 0.93 meq O_2_/kg lipids) and TBARS (from 0.03 to 1.26 mg MDA/kg sample) were found in red tilapia (*Oreochromis niloticus × Tilapia mosambicus*) after 5 months of freezing at −18 °C [25]. The same trend was observed when different fish species were kept frozen at −18 °C for several months, such as red carp (*Cyprinus carpio*) for 9 months [26], rainbow trout (*Oncorhynchus mykiss*) for 10 months [27], and Atlantic mackerel (*Scomber scombrus*) for 12 months [9]. Similar levels of primary and secondary oxidation products were found in Atlantic mackerel when stored at −18 or −25 °C for 12 months [9]. Freezing temperature of −20 °C also resulted in significantly higher PV and TBARS values after 18 months of storage in hoki (*Macruronus novaezelandiae*) and saithe (*Pollachius virens*) [28].

Other authors carried out a study with Atlantic hake (*Merluccius hubbsi*) after 4 months of storage at −18 °C, and although no peroxides or TBARS were measured, they considered the increase in cholesterol oxides (from 16.5 to 149.0 µg/100 g dw) as a marker of lipid deterioration in fish muscle [29].

Increased levels of primary and secondary lipid oxidation products were also found in frozen fish at −30 °C after long-term storage (1 year or more) compared to fresh fish, as reported for blue whiting (*Micromesistius poutassou*) [30], cod (*Gadus morhua*), haddock (*Melanogrammus aeglefinus*) [31], hoki (*Macruronus novaezelandiae*), and saithe (*Pollachius virens*) [28,32]. However, a beneficial effect of long-term frozen storage was found in fillets of both species at −30 °C vs. −20 °C due to the preservation of phospholipids and reduced formation of FFA, hydroperoxides, and secondary oxidation products [28].

Evidence shows that lipid hydrolysis is reduced at lower freezing temperatures due to the inhibition of the enzymatic activity in fish tissues. In this sense, a lower lipase activity was reported in horse mackerel (*Trachurus trachurus*) stored at −80 °C than at −20 °C [7], and a delayed lipid oxidation was observed in anchovy (*Engraulis encrasicholus*) at −80 °C compared to higher storage temperatures (−40 and −20 °C) [23].

A significant increase in lipid oxidation has been reported in refrigerated fish (0–4 °C) at shorter storage times. For example, tuna (*Thunnus thynnus* L.) stored at 0 °C for 18 days increased its TBARS value from 0.34 to 1 mg MDA/kg lipids [33], and sardine (*Sardinella gibbosa*) showed higher values of PV (from 5 to 30 meq O_2_/kg) and TBARS (from 17 to 600 mg MDA/kg) after 15 days storage at 4 °C [16]. Other authors reported a decrease of PV in catfish (*Arius maculatus*) after 9 days of storage at 4 °C, although they attributed this fact to the conversion of peroxides into secondary oxidation products even though such compounds were not measured [34].

The degree of unsaturation of FA contained in fish lipids is a key factor regarding the oxidative stability of such lipids: the higher the unsaturation degree, the more prone to oxidation are those PUFA [35]. In this sense, a decrease of PUFA and monounsaturated FA (MUFA) levels and an increase of saturated FA (SFA) are generally observed in fish lipids after refrigerated or frozen storage, regardless of the temperature and time of storage (Table 1). The decrease of PUFA levels, especially EPA and DHA, is usually reflected in an increase of oxidation products; this is expected as such FA are more sensitive to oxidative degradation than other FA with a lower unsaturation degree. This trend has been reported in many fish species, including *A. maculatus*, *C. carpio* [34], *C. dussumieri* [24], *D. acuta*, *E. affinis*, *S. commerson*, *S. guttatus*, *T. tonggol* [24,36], *L. aurata*, *R. frisii kutum*, *S. lucioperca* [37], *S. aurita, M. merluccius* [38], *M. hubbsi* [29], *O. mykiss* [27,39], *R. canadum* [40], *S. brasiliensis* [41], *O. niloticus x T. mosambicus* [25], *M. novaezelandiae*, and *P. virens* [28]. Generally, greater PUFA losses were found in samples stored at higher temperatures (for instance, −20 °C instead of −30 °C), and DHA was more sensitive to degradative oxidation than EPA, probably due to its higher unsaturation degree. However, in certain cases no decrease of PUFA levels was reported after chilled or frozen storage of fish, such as in *S. scombrus* stored at −18, −27, and −28 °C for 12 months or at 4 °C for 9 days [9,42], or in *S. aurata* stored at 4 °C for 7 days [43]. This different trend could be explained by the variable concentration of compounds with antioxidant activity such as tocopherols, which are naturally present in fish and whose amount depends on factors such as fish diet, season, age, and size [44]. Furthermore, the antioxidant enzymes available in frozen fish tissues, mainly superoxide dismutase, catalase, and glutathione peroxidase, may be active even at freezing temperatures, as it has been shown in animal tissues, thus preventing PUFA oxidation [45,46].

The effect of the joint use of low temperature storage and other preservation techniques on the lipid quality of fish will be reviewed in the following paragraphs.

**Table 1:** Changes of fatty acid profile and lipid quality indexes of several fish species after low temperature storage.

Species	Storage Temperature and Time	Variation of Fatty Acid Profile (Beginning to End of Storage)	Lipid Quality Indexes	Publication Year	Main Findings	Reference
*Arius maculatus* (catfish)	4 °C 9 days	SFA: 46.7 to 49.3 g/100 g oil MUFA: 24.1 to 34.6 g/100 g oil PUFA: 25.4 to 16.4 g/100 g oil EPA: 6.6 to 3.4 g/100 g oil DHA: 5.9 to 3.2 g/100 g oil	PV: 72 to 32 meq O_2_/kg lipids	2017	Lipid oxidation was observed in the storage conditions. EPA and DHA were the most affected PUFA.	[34]
*Carcharhinus dussumieri* (white cheek shark)	−18 °C 6 months	SFA: 43.7 to 57.6 g/100 g oil MUFA: 28.8 to 25.6 g/100 g oil PUFA: 19.9 to 15.1 g/100 g oil EPA: 1.3 to 0.0 g/100 g oil DHA: 11.5 to 7.6 g/100 g oil	FFA: 1.1 to 8.4% PV: 0.2 to 4.4 meq O_2_/kg lipids TBARS: 0.04 to 0.07 mg MDA/kg fillet	2009	Hydrolysis rate and primary and secondary oxidation products increased. EPA and DHA significantly decreased.	[24]
*Clupeonella cultiventris caspia* (common kilka)	−24 °C 6 months	SFA: 36.9 to 45.1% of total FA MUFA: 43.7 to 41.6% of total FA PUFA: 19.4 to 13.8% of total FA EPA: 5.7 to 3.9% of total FA DHA: 9.9 to 6.4% of total FA	Not reported	2010	Significant changes on FA profile after the storage time. Lower nutritional quality by the increase of SFA and decrease of PUFA.	[37]
*Cyprinus carpio* (red carp)	−18 °C 270 days	SFA: 38.4 to 43.1 g/100 g oil MUFA: 32.9 to 41.6 g/100 g oil PUFA: 22.4 to 10.9 g/100 g oil EPA: 4.1 to 2.0 g/100 g oil DHA: 3.0 to 1.8 g/100 g oil	FFA: 1.35 to 8.06% PV: 3.77 to 18.62 meq O_2_/kg lipids	2019	Loss of nutritional quality was found at the end of the study. Lipid quality is retained with storage time < 3 months at −18 °C.	[26]
*Cyprinus carpio* (common carp)	−24 °C 6 months	SFA: 36.0 to 41.0% of total FA MUFA: 48.2 to 47.2% of total FA PUFA: 15.8 to 11.3 % of total FA EPA: 5.3 to 3.3 % of total FA DHA: 6.2 to 4.1% of total FA	Not reported	2010	Significant changes on FA profile after the storage time. Lower nutritional quality by the increase of SFA and decrease of PUFA.	[37]
*Dussumieria acuta* (rainbow sardine)	−18 °C 6 months	SFA: 16.8 to 3.9 mg/g sample MUFA: 3.4 to 0.6 mg/g sample PUFA: 15.3 to 3.1 mg/g sample EPA: 3.4 to 0.6 mg/g sample DHA: 10.2 to 2.1 mg/g sample	Not reported	2014	FA profiles were significantly affected by frozen storage, with significant losses of nutritionally relevant EPA and DHA.	[36]
*Engraulis encrasicholus* (anchovy)	−20 °C 6 months	Not reported	FFA: 1.33 to 4.83% PV: 2.32 to 39.95 meq O_2_/kg TBARS: 1.53 to 13.32 mg MDA/kg	2014	Hydrolysis rate and primary and secondary oxidation products fignificantly increased after freezing at −20 °C for 6 months.	[23]
*Engraulis encrasicholus* (anchovy)	−40 °C 6 months	Not reported	FFA: 1.33 to 4.86% PV: 2.32 to 28.48 meq O_2_/kg TBARS: 1.53 to 12.88 mg MDA/kg	2014	The increase of primary and secondary oxidation products was less pronounced than at −20 °C.	[23]
*Engraulis encrasicholus* (anchovy)	−80 °C 6 months	Not reported	FFA: 1.33 to 6.03% PV: 2.32 to 22.61 meq O_2_/kg TBARS: 1.53 to 9.04 mg MDA/kg	2014	Freezing at −80 °C preserved lipid quality more efficiently than at −20 or −40 °C, but still significant increases of PV and TBARS were observed.	[23]
*Euthynnus affinis* (kawakawa)	−18 °C 6 months	SFA: 6.9 to 3.7 mg/g sample MUFA: 1.9 to 1.2 mg/g sample PUFA: 7.5 to 0.7 mg/g sample EPA: 0.9 to 0.0 mg/g sample DHA: 5.5 to 0.6 mg/g sample	Not reported	2014	FA profiles were significantly affected by frozen storage, with significant losses of nutritionally relevant EPA and DHA.	[36]
*Gadus morhua* (cod)	−10 °C 1 year	Not reported	FFA: 7.1 to 48.8% PV: 2.0 to 1.7 meq O_2_/kg lipids TBARS: 0.1 to 0.3 mg MDA/kg sample	1999	A higher hydrolysis degree was observed at −10 °C than at −30 °C. PV significantly increased until month 9 and then sharply decreased.	[31]
*Gadus morhua* (cod)	−30 °C 1 year	Not reported	FFA: 7.1 to 14.3% PV: 2.0 to 7.9 meq O2/kg lipids TBARS: 0.1 to 0.5 mg MDA/kg sample	1999	A lower hydrolysis degree was observed at −30 °C than at −10 °C. Lipid oxidation increased during the study.	[31]
*Liza aurata* (golden grey mullet)	−24 °C 6 months	SFA: 41.1 to 49.9% of total FA MUFA: 44.7–41.1% of total FA PUFA: 14.2–9.3% of total FA EPA: 2.9 to 4.3% of total FA DHA: 3.9 to 2.4% of total FA	Not reported	2010	Significant changes on FA profile after the storage time. Significant PUFA losses, although EPA eas increased after the freezing treatment.	[37]
*Macruronus novaezelandiae* (hoki)	−30 °C 18 months	SFA: 26.4 to 27.4 g/100 g lipids (LM); 26.0 to 25.3 g/100 g lipids (DM) MUFA: 20.2 to 18.0 g/100 g lipids (LM); 40.1 to 38.3 g/100 g lipids (DM) PUFA: 51.5 to 47.9 g/100 g lipids (LM); 30.1 to 30.0 g/100 g lipids (DM) EPA: 6.5 to 6.2 g/100 g lipids (LM); 7.0 to 6.9 g/100 g lipids (DM) DHA: 38.5 to 35.3 g/100 g lipids (LM); 12.4 to 12.6 g/100 g lipids (DM)	Hydroperoxides: LM: ~10 to 35 mmol/kg muscle DM: ~20 to 250 mmol/kg muscle TBARS: LM: ~5 to 12 µmol MDA/kg muscle DM: ~10 to 35 µmol MDA/kg muscle	2014	Fatty acid profile and lipid quality were less affected than freezing at −20 °C for the same species. No significant losses of EPA was found in LM and DM. Significant loss of DHA in LM but not in DM was detected after frozen storage at −30 °C for 18 months.	[28]
*Macruronus novaezelandiae* (hoki)	−20 °C 18 months	SFA: 26.4 to 26.9 g/100 g lipids (LM); 26.0 to 25.2 g/100 g lipids (DM) MUFA: 20.2 to 20.2 g/100 g lipids (LM); 40.1 to 40.8 g/100 g lipids (DM) PUFA: 51.5 to 46.2 g/100 g lipids (LM); 30.1 to 27.9 g/100 g lipids (DM) EPA: 6.5 to 5.9 g/100 g lipids (LM); 7.0 to 6.0 g/100 g lipids (DM) DHA: 38.5 to 31.0 g/100 g lipids (LM); 12.4 to 9.7 g/100 g lipids (DM)	Hydroperoxides: LM: ~10 to 120 mmol/kg muscle DM: ~20 to 320 mmol/kg muscle TBARS: LM: ~5 to 14 µmol MDA/kg muscle DM: ~10 to 28 µmol MDA/kg muscle	2014	Fatty acid profile and lipid quality were more affected than freezing at −30 °C for the same species. Significant losses of EPA and DHA were found in LM and DM.	[28]
*Melanogrammus aeglefinus* (haddock)	−10 °C 1 year	Not reported	FFA: 9.0 to 54.2% PV: 1.8 to 0.8 meq O_2_/kg lipids TBARS: 0.3 to 0.3 mg MDA/kg sample	1999	A higher hydrolysis degree was observed at −10 °C than at −30 °C. PV significantly increased until month 9 and then sharply decreased.	[31]
*Melanogrammus aeglefinus* (haddock)	−30 °C 1 year	Not reported	FFA: 9.0 to 21.1% PV: 1.8 to 8.5 meq O_2_/kg lipids TBARS: 0.3 to 0.4 mg MDA/kg sample	1999	A lower hydrolysis degree was observed at −30 °C than at −10 °C. Lipid oxidation (particularly PV) increased during the study.	[31]
*Merluccius hubbsi* (Atlantic hake)	−18 °C 4 months	SFA: 21.8 to 21.6 g/100 g oil MUFA: 45.2 to 36.7 g/100 g oil PUFA: 29.5 to 14.7 g/100 g oil EPA: 6.8 to 4.0 g/100 g oil DHA: 13.0 to 7.9 g/100 g oil	Cholesterol oxides: 16.5 to 149.0 µg/100 g dry weight	2008	Lipid nutritional quality decreased after frozen storage (−18 °C) for 4 months with significant losses of EPA and DHA and an increased value of cholesterol oxides.	[29]
*Merluccius merluccius* (hake)	−30 °C 1 month	SFA: 45.9 to 59.6% of total FA MUFA: 13.6 to 17.7% of total FA PUFA: 40.5 to 22.8% of total FA EPA: 7.4 to 3.0% of total FA DHA: 24.2 to 8.1% of total FA	Not reported	2013	Increased proportion of SFA and decreased levels of PUFA (particularly EPA and DHA) led to a loss of nutritional quality after 1 month of frozen storage.	[38]
*Micromesistius poutassou* (blue whiting)	−30 °C 1 year	Not reported	FFA: 5.0 to 14.1% PV: 3.1 to 9.3 meq O_2_/kg lipids TBARS: 0.3 to 0.6 mg MDA/kg simple	1999	Significantly increased values for lipid hydrolysis and lipid oxidation were found after frozen storage at −30 °C.	[30]
*Micromesistius poutassou* (blue whiting)	−10 °C 1 year	Not reported	FFA: 5.0 to 55.8% PV: 3.1 to 6.5 meq O_2_/kg lipids TBARS: 0.3 to 0.2 mg MDA/kg sample	1999	Higher hydrolysis rate compared to the freezing at −30 °C. Lipid indexes were more favorable, which could be due to a lack of accuracy of measurements.	[30]
*Oncorhynchus mykiss* (rainbow trout)	−15 °C 45 days	SFA: 24.7 to 32.9% of total FA MUFA: 31.2 to 32.7% of total FA PUFA: 44.4 to 34.1% of total FA EPA: 5.64 to 4.88% of total FA DHA: 17.28 to 11.08% of total FA	Not reported	2014	A significant decrease of PUFA, particularly EPA and DHA, was found during frozen storage for 45 days.	[39]
*Oncorhynchus mykiss* (rainbow trout)	−15 °C 90 days	SFA: 24.7 to 32.7% of total FA MUFA: 31.2 to 34.1% of total FA PUFA: 44.4 to 33.2% of total FA EPA: 5.64 to 4.13% of total FA DHA: 17.28 to 10.59% of total FA	Not reported	2014	No significant effect of time (45 vs. 90 days) was found regarding losses of EPA and DHA.	[39]
*Oncorhynchus mykiss* (rainbow trout)	−18 °C 10 months	SFA: 26.3 to 36.6% of total FA MUFA: 33.0 to 29.0% of total FA PUFA: 23.8 to 17.2% of total FA EPA: 5.8 to 2.3% of total FA DHA: 7.6 to 6.4% of total FA	PV: 0.10 to 0.49 meq O_2_/kg lipids TBARS: 0.17 to 0.78 mg MDA/kg fillet	2014	Nutritional value of fish lipids was decreased after frozen storage, although still in the acceptable range for human consumption.	[27]
*Oreochromis niloticus × Tilapia mosambicus* (red tilapia)	−18 °C 150 days	SFA: 27.1 to 30.1% total lipids MUFA: 39.0 to 44.5% total lipids PUFA: 33.5 to 24.8% total lipids EPA: 0.63 to 0.30% total lipids DHA: 6.95 to 3.42% total lipids	PV: 0.02 to 0.93 meq O_2_/kg lipids TBARS: 0.03 to 1.26 mg MDA/kg sample	2013	Lipid nutritional quality decreased after frozen storage but PV and TBARS were not above the acceptable range for human consumption.	[25]
*Pollachius virens* (saithe)	−30 °C 18 months	SFA: 23.5 to 23.3 g/100 g lipids (LM); 20.5 to 21.0 g/100 g lipids (DM) MUFA: 15.1 to 14.4 g/100 g lipids (LM); 20.7 to 18.8 g/100 g lipids (DM) PUFA: 59.1 to 56.9 g/100 g lipids (LM); 55.0 to 54.6 g/100 g lipids (DM) EPA: 13.4 to 11.8 g/100 g lipids (LM); 9.3 to 9.3 g/100 g lipids (DM) DHA: 40.0 to 39.4 g/100 g lipids (LM); 38.8 to 38.1 g/100 g lipids (DM)	Hydroperoxides: LM: ~10 to 20 mmol/kg muscle DM: ~10 to 20 mmol/kg muscle TBARS: LM: ~6 to 12 µmol MDA/kg muscle DM: ~6 to 20 µmol MDA/kg muscle	2014	Fatty acid profile and lipid quality were less affected than freezing at −20 °C for the same species. A significant loss of EPA was detected in LM but not in DM. No significant loss of DHA was detected in LM or DM after frozen storage at −30 °C for 18 months.	[28]
*Pollachius virens* (saithe)	−20 °C 18 months	SFA: 23.5 to 23.7 g/100 g lipids (LM); 20.5 to 20.4 g/100 g lipids (DM) MUFA: 15.1 to 15.3 g/100 g lipids (LM); 20.7 to 20.8 g/100 g lipids (DM) PUFA: 59.1 to 55.5 g/100 g lipids (LM); 55.0 to 53.0 g/100 g lipids (DM) EPA: 13.4 to 11.0 g/100 g lipids (LM); 9.3 to 7.9 g/100 g lipids (DM) DHA: 40.0 to 33.6 g/100 g lipids (LM); 38.8 to 36.4 g/100 g lipids (DM)	Hydroperoxides: LM: ~10 to 35 mmol/kg muscle DM: ~10 to 20 mmol/kg muscle TBARS: LM: ~6 to 20 µmol MDA/kg muscle DM: ~6 to 30 µmol MDA/kg muscle	2014	Fatty acid profile and lipid quality were more affected than freezing at −30 °C for the same species. Significant losses of EPA and DHA were found in LM and DM.	[28]
*Rachycentron canadum* (cobia)	−18 °C 6 months	SFA: 46.1 to 51.3 g/100 g lipids MUFA: 33.7 to 26.3 g/100 g lipids PUFA: 15.4 to 10.8 g/100 g lipids EPA: 1.8 to 0.8 g/100 g lipids DHA: 5.8 to 3.4 g/100 g lipids	Not reported	2012	FA profile was modified by frozen storage and a marked decrease of PUFA, particularly EPA and DHA, was found.	[40]
*Rutilus frisii kutum* (Caspian kutum)	−24 °C 6 months	SFA: 29.0 to 37.1% of total FA MUFA: 56.3 to 53.6% of total FA PUFA: 14.8 to 9.3% of total FA EPA: 4.6 to 2.9% of total FA DHA: 7.1 to 3.9% of total FA	Not reported	2010	Significant changes on FA profile after the storage time. Lower nutritional quality by the increase of SFA and decrease of PUFA, including EPA and DHA.	[37]
*Sander lucioperca* (pike perch)	−24 °C 6 months	SFA: 36.0 to 45.3% of total FA MUFA: 41.0 to 39.3% of total FA PUFA: 23.0 to 15.3% of total FA EPA: 3.2 to 2.5% of total FA DHA: 11.4 to 7.4% of total FA	Not reported	2010	Significant changes on FA profile after the storage time. Lower nutritional quality by the increase of SFA and decrease of PUFA, including EPA and DHA.	[37]
*Sardinella aurita* (sardinella)	−30 °C 1 month	SFA: 49.2 to 53.4% of total FA MUFA: 12.0 to 15.2% of total FA PUFA: 38.8 to 31.4% of total FA EPA: 8.5 to 3.0% of total FA DHA: 23.1 to 11.0% of total FA	Not reported	2013	Increased proportion of SFA and decreased levels of PUFA (particularly EPA and DHA) led to a loss of nutritional quality.	[38]
*Sardinella brasiliensis* (sardine)	−18 °C 120 days	SFA: 16.5 to 16.9 g/100 g lipids MUFA: 21.1 to 16.1 g/100 g lipids PUFA: 38.0 to 27.1 g/100 g lipids EPA: 11.4 to 7.8 g/100 g lipids DHA: 16.7 to 12.2 g/100 g lipids	Cholesterol oxides: 19.4 to 115.2 µg/g dry weight	2008	Lipid nutritional quality decreased with significant losses of EPA and DHA and increased values of cholesterol oxides.	[41]
*Sardinella gibbosa* (sardine)	4 °C (ice) 15 days	SFA: 45.9 to 46.9 g/100 g oil MUFA: 16.7 to 15.1 g/100 g oil PUFA: 35.7 to 32.9 g/100 g oil EPA: 6.14 to 4.96 g/100 g oil DHA: 19.7 to 18.5 g/100 g oil	PV: 5 to 30 meq O_2_/kg lipids TBARS: 17 to 600 mg MDA/kg sample	2006	Lipid oxidation measured by PV and TBARS was remarkable under chilled storage, as well as losses of EPA and DHA.	[16]
*Scomber scombrus* (Atlantic mackerel)	−25 °C 12 months	SFA: 25.5 to 25.0 g/100 g lipids MUFA: 32.9 to 29.4 g/100 g lipids PUFA: 33.8 to 36.3 g/100 g lipids EPA: 8.7 to 9.2 g/100 g lipids DHA: 11.2 to 12.0 g/100 g lipids	Hydroperoxides: ~0.09 to 0.35 µmol/g muscle TBARS: ~0.5 to 1.0 µmol MDA/g muscle	2016	EPA and DHA were more preserved at −25 °C than at −18 °C. No major differences in lipid oxidation indices were observed.	[9]
*Scomber scombrus* (Atlantic mackerel)	−18 °C 12 months	SFA: 25.5 to 21.7 g/100 g lipids MUFA: 32.9 to 35.5 g/100 g lipids PUFA: 33.8 to 32.7 g/100 g lipids EPA: 8.7 to 7.9 g/100 g lipids DHA: 11.2 to 11.3 g/100 g lipids	Hydroperoxides: ~0.09 to 0.38 µmol/g muscle TBARS: ~0.5 to 1.1 µmol MDA/g muscle	2016	EPA and DHA was less preserved at −18 °C than at −25 °C. No major differences in lipid oxidation indices were observed.	[9]
*Scomber scombrus* (Atlantic mackerel)	4 °C (vacuum packed) 9 days	SFA: 23.7 to 24.2% of total FA MUFA: 47.8 to 47.5% of total FA PUFA: 28.2 to 28.0% of total FA EPA: 7.2 to 7.1% of total FA DHA: 10.0 to 10.1% of total FA	TBARS: ~0.5 mmol MDA/kg muscle	2018	FA profile was maintained after 9 days of chilled storage at 9 °C, but lipolysis increased compared to frozen storage (−27 °C) for 1 year.	[42]
*Scomber scombrus* (Atlantic mackerel)	−27 °C (vacuum packed) 12 months	SFA: 23.7 to 21.5% of total FA MUFA: 47.8 to 45.6% of total FA PUFA: 28.2 to 29.0% of total FA EPA: 7.2 to 7.1% of total FA DHA: 10.0 to 11.0% of total FA	TBARS: ~0.2 mmol MDA/kg muscle	2018	FA profile was maintained after 1 year of frozen storage at −27 °C.	[42]
*Sparus aurata* (sea bream)	4 °C 7 days	SFA: 20.5 to 20.7% of total FA MUFA: 19.4 to 22.1% of total FA PUFA: 45.4 to 44.6% of total FA EPA: 0.4 to 0.5% of total FA DHA: 23.8 to 22.0% of total FA	Not reported	2007	FA profile was maintained after chilling at 4 °C for a short time (1 week).	[43]
*Scomberomorus commerson* (Spanish mackerel)	−18 °C 6 months	SFA: 21.0 to 10.2 mg/g sample MUFA: 7.0 to 4.8 mg/g sample PUFA: 12.9 to 4.6 mg/g sample EPA: 1.6 to 0.3 mg/g sample DHA: 7.7 to 3.3 mg/g sample	Not reported	2014	FA profiles were significantly affected by frozen storage, with significant losses of nutritionally relevant EPA and DHA.	[36]
*Scomberomorus commerson* (Spanish mackerel)	−18 °C 6 months	SFA: 44.2 to 60.0 g/100 g lipids MUFA: 27.5 to 25.7 g/100 g lipids PUFA: 22.2 to 13.1 g/100 g lipids EPA: 3.9 to 1.8 g/100 g lipids DHA: 13.0 to 7.2 g/100 g lipids	FFA: 3.2 to 5.7% PV: 2.3 to 15.4 meq O_2_/kg lipids TBARS: 0.04–0.10 mg MDA/kg fillet	2009	FA profiles were significantly affected by frozen storage, with significant losses of both EPA and DHA. A marked increase of lipid hydrolysis and oxidation rates were observed.	[24]
*Scomberomorus guttatus* (King mackerel)	−18 °C 6 months	SFA: 2.5 to 1.0 mg/g sample MUFA: 0.7 to 0.1 mg/g sample PUFA: 4.0 to 0.3 mg/g sample EPA: 0.4 to 0.0 mg/g sample DHA: 3.0 to 0.2 mg/g sample	Not reported	2014	FA profiles were significantly affected by frozen storage, with significant losses of nutritionally relevant EPA and DHA.	[36]
*Thunnus thynnus* L. (tuna)	0 °C 18 days	SFA: 30.7 to 34.2% of total FA MUFA: 20.3 to 21.3% of total FA PUFA: 41.3 to 38.6% of total FA EPA: 7.5 to 7.8% of total FA DHA: 26.5 to 23.7% of total FA	TBARS: 0.34 to 1 mg MDA/kg	2008	Significant SFA increase, PUFA decrease, and TBARS increase was observed after storage at 0 °C for 18 days.	[33]
*Thunnus tonggol* (longtail tuna)	−18 °C 6 months	SFA: 4.1–2.1 mg/g sample MUFA: 1.2–0.3 mg/g sample PUFA: 4.1–1.6 mg/g sample EPA: 0.5 to 0.1 mg/g sample DHA: 2.9 to 1.4 mg/g sample	Not reported	2014	FA profiles were significantly affected by frozen storage, with significant losses of nutritionally relevant EPA and DHA.	[36]

DM: dark muscle; FFA: free fatty acids; MDA: malondialdehyde; MUFA: monounsaturated fatty acids; LM: light muscle; PUFA: polyunsaturated fatty acids; PV: peroxide value; SFA: saturated fatty acids; TBARS: thiobarbituric acid reactive substances.

### 3.2. Use of Natural Preservatives

The use of natural antioxidant compounds is a strategy to protect seafood from free radical damage during low temperature storage. Supplementing fish with antioxidants (tocopherols, ascorbic acid, phenols, carotenoids, antioxidant enzymes, chitosan, etc.), either in the diet or directly in processed fish products, can be an effective approach to minimize or retard the lipid peroxidation processes, prevent or delay the production of hazardous oxidation products, and maintain nutritional quality during the processing and the post-mortem storage of fish products [47,48] (Table 2). In fact, antioxidants are essential substances for the preservation of seafood due to their scavenging activity against free radicals [15]. In recent years, the food industry has prioritized the use of natural antioxidants as alternatives to synthetic antioxidants such as butylated hydroxytoluene (BHT), butylated hydroxyanisole (BHA), propyl gallate (PG) and ethoxyquin (EQ) due to the consumers’ preference for natural antioxidants and the concerns raised about the possible adverse health effects of some of the synthetic compounds [49,50].

Chitosan is a natural non-toxic biopolymer resulting from partial or total deacetylation of chitin, which has applications as food preservative because of its antimicrobial activity and film-forming properties [51]. Chitosan addition has shown good results to retain lipid quality by retarding oxidation processes during cold storage (−3 to 4 °C) of fish species such as Nile tilapia (*Oreochromis niloticus*) fillets, pangasius (*Pangasianodon hypophthalmus*) surimi, or silver carp (*Hypophthalmichthys molitrix*) slices [52]. Coating with chitosan combined with extracts of phenolic compounds led to a decrease of primary and secondary lipid oxidation products in fishes such as red drum (*Sciaenops ocellatus*) or large yellow croaker (*Pseudosciaena crocea*) [52]. Another study showed a significant decrease of secondary lipid oxidation products (TBARS value) after up to 12 days of refrigerated storage (4 °C) of rainbow trout (*Oncorhynchus mykiss*) fillets in those samples coated with either chitosan or a chitosan plus a lactoperoxidase-containing system (4.42 and 4.48 mg MDA/kg, respectively) compared to the control group (no coating) (5.01 mg MDA/kg) [51]. Alginate has also been used as coating agent in fish fillets, either alone or combined with antioxidant additives. In a study, rainbow trout (*O. mykiss*) fillets, to which tannic acid or quebracho extract (condensed tannins) was added as antioxidants, were stored at 4 °C for 15 days with or without alginate coating, and it was observed that TBARS values were significantly lower using alginate [53]. This fact can be explained because of the alginate layer preventing oxygen diffusion.

Phenolic compounds are known for their antioxidant activity, which is exerted through various pathways such as free radical scavenging, quenching of reactive oxygen species, inhibition of oxidative enzymes, and chelation of transition metals or interaction with biomembranes. Beyond the presence of polyphenols in natural foods, the interest in food technology lies in the possibility of extracting them from their respective sources and subsequently adding them to some foods for their antioxidant properties [54]. The antioxidant activity of phenolic compounds isolated from by-products of the viticulture industry was tested on minced flesh of Atlantic mackerel (*S. scombrus*) frozen at −10 °C, and a significant inhibition of peroxides and aldehydes (primary and secondary lipid oxidation products, respectively) was reported from 39 to up to 83 days of storage. Furthermore, although a loss of DHA was found in both control and treated samples, the decrease was less pronounced after 12 weeks of storage in those samples treated with phenols [55]. Many other experimental results have proven the ability of plant-based phenolic compounds to retard lipid oxidation in frozen or refrigerated fish and fish products such as silver carp (*Hypophthalmicthys molitrix*), mackerel (*Scomber scombrus*), common carp (*Cyprinus carpio*), and coho salmon (*Oncorhynchus kisutch*) [56,57].

Comparative studies on the effects of dietary α-tocopherol acetate or post-slaughter addition on the lipid stability of fish during storage have shown that post-mortem addition of α-tocopherol improved the lipid stability of tilapia burgers during frozen storage at −18 °C for 90 days [58]. In fish fillets, aqueous solutions of ascorbic acid and citric acid have showed effective antioxidant properties [7]. Ascorbic acid is a potent antioxidant that acts as a metal chelator and oxygen scavenger agent and has a direct synergistic relationship with other antioxidants. This compound is commonly used to extend the shelf life of canned and processed food (oil fish mince and fillets) without limiting consumption. Moreover, citric acid is another well-known natural preservative in the food industry that plays an important role in lipid oxidation and enzyme inhibition, especially in frozen fillets [59]. It was also found that α-tocopherol treatment maintains lipid stability in fish fillets [60].

Furthermore, extracts and essential oils of different plant species such as thyme, ginseng, sage, or rosemary, alone or in combination with other compounds such as tocopherols, have shown promising results in maintaining nutritional quality and preventing lipid oxidation in marine edible products [61]. These antioxidant properties are mainly attributed to their high phenolic content [48]. For instance, Indian mackerel fillets were treated with mint or citrus extract solutions at 0.5 and 1% *w/v*, respectively, for 30 min and then stored at −20 °C for up to 8 months together with untreated (control) samples [62]. The content of FFA was increased during the experiment in all cases, and the values were significantly higher in the control samples (5.76% oleic acid) than in the treated samples (4.89 and 5.24% oleic acid for mint and citrus treated samples, respectively) at the end of the storage period, with no significant differences between them. PV also increased in all samples during the experiment, but its values, as well as the peroxide rate production, were lower in treated samples than in controls, and this inhibition of peroxide production was found particularly in samples treated with the mint extract. TBARS showed a similar trend to PV, with a significant increase from 0.226 mg MDA/kg sample to 1.334, 1.372, and 1.217 mg MDA/kg sample for control, mint, and citrus treated samples, respectively. TBARS levels were significantly lower in the mint-treated samples than in the citrus-treated samples. According to the results of the study, the treatment of the fish fillets with mint and citrus extracts may be an effective strategy to minimize lipid hydrolysis and oxidation [62].

Another source of bioactive compounds are marine algae and microalgae; they are multicomponent antioxidant systems due to the presence of a high amount of bioactive molecules such as pigments, polyphenols, phycobiliproteins, minerals, and vitamins that give them a powerful antioxidant and immunomodulatory activity, which is doubled due to the interactions between its components. The potential effects of these treatments are related to delayed lipid oxidation, inhibited microbial growth, and improved sensory properties [48,63]. The value of microalgae as a source of natural antioxidants is enhanced by the relative ease of purification of the target compounds. Recent research has shown the efficacy of these compounds. For example, a study reported a decrease in secondary peroxidation during 170 days at −40 °C using seaweed extracts (cochayuyo, sea lettuce, ulte, and red luche) in the covering liquid of canned salmon compared to the control sample [64]. Other authors showed inhibition of lipid oxidation by adding an extract of Icelandic brown seaweed (*Fucus vesiculosus*) to cod bone mince protein hydrolysates [65]. Similarly, brown seaweed extracts caused a strong inhibition of hemoglobin-mediated lipid oxidation in a fish muscle model system [66]. An efficient antioxidant activity of extracts of two microalgae species (*Clorella vulgaris* and *Arthorspira platensis*) was also observed on rainbow trout fillets at two different concentrations (0.05 and 0.1%) [67]. Aqueous extracts of the microalgae *Crassiphycus corneus*, *Ulva ohnoi*, *Arthrospira platensis*, and *Haematococcus pluvialis* were tested as additives for the preservation of rainbow trout fillets, showing that their antioxidant and antimicrobial properties were able to extend the shelf life of the fish fillets compared to the ascorbic acid and untreated controls [68].

### 3.3. High Hydrostatic Pressure

High hydrostatic pressure (HHP) has been identified as a potential technology to inhibit the growth of microorganisms and to inactivate hydrolytic enzymes (lipases, peroxidases, phospholipases, etc) in foods, thus preserving their organoleptic and nutritional quality [69]. HHP has been studied as a pretreatment of seafood prior to freezing or chilling to verify its effect on lipid quality (Table 2). HHP can reduce the negative effects of water crystallization on food and has been tested during the freezing processes of several fish species [70]. However, denaturation of iron-bound proteins induced by HHP could release free iron, which may promote lipid oxidation [71].

A study compared the effect of an HHP treatment at different pressures (150, 300, and 450 MPa) and times (0, 2.5, and 5 min) versus a control group without HHP on mackerel (*S. scombrus*) muscle undergoing frozen storage (−10 °C) for 3 months [71]. It was concluded that the HHP treatment had little effect on PV, and TBARS values showed no clear trend between treatment and control groups at any of the assayed combinations of pressure and time. The higher pressure (450 MPa) and time (5 min) treatments resulted in a significant inhibition of lipid hydrolysis in fish tissues, thus preventing the increase in FFA during frozen storage. Although FFA is not a direct marker of lipid oxidation, the oxidation rate of FFA is higher than that of fatty acids esterified in acylglycerols [72], and therefore a high amount of FFA in a tissue may increase the likelihood that the lipids contained in that tissue will be readily oxidized during extended storage periods.

Similar results were found for muscle lipids of farmed coho salmon (*Oncorhynchus kisutch*) after 20 days of refrigerated storage (4 °C) for 20 days with a HHP pretreatment at 135, 170, and 200 MPa for 30 sec. Although no significant effect regarding lipid hydrolysis and FFA formation was observed between the control group (without pressure pretreatment) and the tissues tested at 135 and 170 MPa, those subjected at the highest pressure (200 MPa) showed a delay in FFA formation compared to the control group (244.3 and 328.9 mg FFA/100 g muscle respectively) [73].

In this line, other authors reported a delay of lipid hydrolysis after frozen storage (−18 °C for 9 months) of sardine (*Sardina pilchardus*) previously subjected to a HHP treatment between 125 and 200 MPa, especially after the more intense pressure treatment (200 MPa) [74]. Significantly lower PV were found in frozen samples pretreated with HHP compared to the control after 3 months of frozen storage, but a general trend could not be established for the entire duration of the experiment. Furthermore, no clear relationship was found between TBARS values and HHP-treated samples, regardless of the intensity of the applied pressure.

Other work compared the degree of lipid oxidation of minced mackerel (*S. scombrus*) treated with HHP (200 and 300 MPa for 5 min) and then stored at −30 °C for 3 weeks. It was found that both PV and TBARS were the highest and lowest in the 200 and 300 MPa treatments, respectively, with the control sample (without HHP) showing intermediate values for both parameters [75]. According to these authors, the reason for such finding is that 200 MPa is not high enough to inhibit pro-oxidative endogenous enzymes in the fish material although pro-oxidative iron is released from protein denaturation, whereas the treatment at 300 MPa is efficient to inactivate these pro-oxidative endogenous enzymes, thus reducing the lipid oxidation rate.

### 3.4. Packaging

Vacuum and modified atmosphere packaging techniques combined with refrigeration or freezing have been found to be appropriate methods for preserving seafood because they reduce the amount of oxygen (which acts as a source of reactive oxygen species) and reduce the amount of water in the environment (lipases are more mobile in aqueous environments) [12,76]. The use of vacuum packaging to remove oxygen has been investigated as a means of delaying lipid oxidation during frozen storage of fish (Table 2). For instance, the effectiveness of vacuum packaging in preventing lipid degradation along with frozen storage was assessed on coho salmon (*Oncorhynchus kisutch*) flesh stored at −18 °C in polyethylene bags for 18 months under four different conditions: (i) no vacuum (control); (ii) vacuum; (iii) vacuum plus a phenol-containing film at a concentration of 48 µg p-coumaric + ferulic acids/dm^2^; (iv) vacuum plus a phenol-containing film at a concentration of 128 µg p-coumaric + ferulic acids/dm^2^ [77]. The PV values in the control group at the end of the storage period were significantly higher than in the three groups using vacuum, indicating an inhibitory effect on primary lipid oxidation with the use of vacuum. The use of the phenol-rich film resulted in a non-significant decrease in PV compared to the use of vacuum alone. Secondary lipid oxidation, as measured by the anisidine index (AI), showed a similar trend to PV, as a significantly higher value was found in the control group compared to the three other groups where vacuum was applied prior to frozen storage. Thus, it was concluded that an inhibitory effect on lipid oxidation was exerted using vacuum packaging, although the additional use of a polyphenol-rich film did not show a significant improvement.

Another study reported a significant decrease in FFA in Russian sturgeon (*Acipenser gueldenstaedtii*) flesh after 360 days of storage at −18 °C in vacuum-sealed samples compared to samples stored without vacuum, thus showing that vacuum was able to delay the lipolysis of acylglycerols (TAG and PL) [78]. These authors also found that the decrease in total PUFA (30% of total FA before freezing) was lower in frozen fish stored under vacuum (PUFA: 29.89% of total FA) than in samples stored without vacuum (PUFA: 28.04% of total FA). Regarding DHA losses after freezing, they were significantly lower in vacuum-stored samples than in samples without vacuum (18% and 28% less DHA, respectively, compared to samples before freezing) [78].

Other authors compared lipid quality indices in frozen fillets of yellow-tailed emperor (*Lethrinus atkinsoni*) stored at −18 °C for 40 days with or without vacuum storage [79]. The values of FFA, PV, and TBARS increased from day 0 to day 40 in both groups, but they were significantly lower in the vacuum-stored samples for the three lipid quality indices, suggesting a positive effect of vacuum on the shelf life of the fillet lipids.

### 3.5. Irradiation

Irradiation is a well-known sterilization technology that can be applied to foods to extend their shelf life at doses even higher than 10 kGy, although lipid oxidation has sometimes been reported in irradiated foods [80]. However, the combination of irradiation with other preservation technologies such as freezing can reduce this undesirable effect [80] (Table 2).

The effect of irradiation on lipid quality of vacuum-packed cod (*Gadus morhua*) fillets was assayed at different levels (0, 2, 4, 7, and 10 kGy Electron-beam radiation at room temperature) and then stored at −20 °C for 1 week [81]. Electron-beam (E-beam) irradiation uses ionizing radiation and is characterized by its low cost, high safety, and environmental friendliness [81]. Irradiation did not significantly alter the fat content and FA profiles of fish fillets. Although TBARS levels were significantly higher in all irradiated samples (0.17–0.18 mg MDA/kg) than in the control (0.11 mg MDA/kg), they were far below the level of 5–8 mg MDA/kg considered to be the upper limit for fish acceptability [82].

Patagonian toothfish (*Dissostichus eleginoides*) was frozen and further irradiated with gamma rays (1 and 5 kGy) and stored at −18 °C for 293 days. Irradiation at either intensity did not significantly affect the FA profiles of fish muscle at the end of the storage time compared to unirradiated samples (control) [83].

Tilapia (*Oreochromis* sp.) fillets were irradiated at either 1 or 3 kGy, packed in polystyrene bags and stored at 0 °C for 77 days. PV and TBARS values increased up to 28 days until refrigerated storage and then decreased until the end of the study period [84]. Such data support the fact that irradiation promotes lipid oxidation in fish tissues at short storage times, although the decrease in PV and TBARS values from day 28 to 77 could be explained considering a possible loss of volatile oxidized lipids at longer durations.

### 3.6. Ozonation

The use of ozone as a sanitizer in the food industry, either as a gas or dissolved in water, has been identified as a safe and environmentally friendly alternative to conventional methods of preserving food quality [85]. In addition to its bactericidal effect, ozone has been used in combination with chilled or frozen storage of fish products and the effects on fish lipid quality have been evaluated (Table 2).

The degree of lipid oxidation according to the TBARS values was checked in yellow croaker (*Pseudosciaena crocea*) stored for 21 days under three ice treatments: flake ice (FI), slurry ice (SI), and ozone slurry ice (OSI) [86]. The TBARS values were gradually increased during the storage time and such trend was more pronounced in the FI group compared to the samples treated with the slurry ice (SI and OSI), showing a preventive effect on lipid oxidation in SI and OSI samples. Although OSI was more efficient than SI to inhibit the growth of several microorganisms such as psychrophilic and pseudomonas bacteria, no positive impact on lipid oxidation was shown for OSI versus SI.

Another study explored the influence of different ozone treatments on the degree of lipid oxidation of farmed Atlantic salmon fillets stored at 4 °C for up to 10 days [87]. The variables were the aqueous ozone concentration (1.0 and 1.5 mg/L), the number of passes under spray nozzles (1 to 3), and type of contact (top or top and bottom of fillets). Although TBARS levels increased from 0.35 mg MDA/kg (day 0) to 1.74 mg MDA/kg (day 10), lipid quality was not adversely affected. No significant effect was found among the different ozone treatments performed in this work. However, these authors also examined the effect of the ozone treatments on the production of propanal, which is a secondary oxidation product derived from n-3 PUFA. A significant effect of the aqueous ozone concentration but not of the number of passes under the nozzles was identified, with a 30% higher propanal concentration using 1.0 than 1.5 mg/L of aqueous ozone [87].

The effect of the ozone gas exposure on lipid oxidation was also studied in four different marine species: hake (*Merluccius merluccius*), scald fish (*Arnoglossus laterna*), musky octopus (*Eledone moschata*), and red shrimp (*Aristeus antennatus*). Several cycles and concentrations of ozone were applied according to each species and TBARS values were compared to those of a control group (not exposed to ozone) after storage at 2 °C for 12 days [88]. In all cases other than hake, TBARS values were significantly higher in ozone-treated samples than in control samples at the end of the study period, with hake showing the opposite behaviour. The acceptable TBARS limit was not exceeded in any of the control or treated samples.

### 3.7. Ultrasonication

Ultrasonication technology provides high-frequency acoustic energy that has been shown to be efficient for microbial inactivation in food matrices without significantly affecting their nutritional and organoleptic quality [89]. Ultrasound-assisted freezing (UAF) has been investigated to preserve the lipid quality of fish (Table 2). UAF was compared with air freezing and immersion freezing in common carp (*Cyprinus carpio*) muscle [90]. UAF-treated samples were subjected to ultrasounds (175 W, 30 kHz, 30 s on/30 s off cycle for 9 min) and all samples were stored at −18 °C for 180 days. Although TBARS levels increased over the course of the experiment in all groups compared to the control (day 0, 0.20 mg MDA/kg), they were significantly lower at the end of the experiment in UAF-treated samples (0.37 mg MDA/kg) than in the other two groups (0.43 and 0.48 mg MDA/kg for immersion freezing and air freezing, respectively). The authors explained this fact by the production of small and uniform ice crystals and the possible inhibition of lipid oxidation-promoting enzymes by the ultrasound treatment [90].

In the same line, a recent work explored the effect of UAF versus air freezing (AF) in vacuum-packed flesh of large yellow croaker (*Pseudosciaena crocea*) treated with ultrasounds (25 kHz, 30 sec on/45 sec off) and then stored at −20 °C [91]. After freezing, samples were thawed up to 5 cycles (4 °C for 12 h followed by freezing at −20 °C for 24 h) and TBARS values were calculated. After five freeze-thaw cycles, TBARS values increased 2.70-fold and 10.40-fold in UAF-treated samples and AF-treated samples, respectively, compared to the control, indicating that UAF could prevent lipid oxidation more efficiently than AF.

Conversely, UAF at different power levels (0 to 0.60 W/cm^2^) was also evaluated on fresh grass carp (*Ctenopharyngodon idella*) muscle stored at −18 °C in a cooling solution of water:ethanol (1:1 *v*/*v*) using a frequency of 40 kHz, and the results showed a small but significant increase in TBARS levels (from 0.59 to 0.69 mg MDA/kg) together with the higher power of the ultrasound treatment compared to the control (no sonicated sample) [92]. In particular, the authors observed that ultrasound power higher than 0.48 W/cm^2^ promoted lipid oxidation in frozen grass carp muscle, which could be due to the increased production of reactive oxygen compounds at the highest ultrasound power tested in this work.

The effect of ultrasonic treatments has also been tested in the thawing of frozen fish. In this regard, a study compared three thawing methods: air thawing (AT), water immersion thawing (WIT), and ultrasonic thawing (UT) (28 kHz, no pulse-off) to check the effect on lipid stability of bighead carp (*Aristichthys nobilis*) stored at −18 °C for 30 days [93]. TBARS values were significantly lower in UT and WIT samples compared to AT samples, although no significant differences were found between UT and WIT groups. Another study investigated the effect of a multi-frequency ultrasound treatment (20, 28 and 40 kHz) during thawing using frozen eviscerated large yellow croaker (*Larimichthys crocea*) at −18 °C for 7 days compared to air thawing and flowing water thawing [94]. Although all samples showed a higher lipid oxidation than the control (fresh fish), TBARS measurements showed significantly lower values (*p* < 0.05) in samples subjected to the multi-frequency ultrasound thawing (0.10–0.11 mg MDA/kg) than in air or flowing water thawing (0.14–0.16 mg MDA/kg), indicating a better preservation of the lipid fraction in such samples.

The efficiency of ultrasound in thawing was also evaluated in mirror carp (*Cyprinus carpio*) stored at −18 °C for 7 days, and it was found that ultrasound thawing resulted in significantly lower TBARS values than air thawing, and that lipid oxidation was more inhibited when ultrasound thawing was performed in saline solutions (0.05 to 0.20% salt) [95]. However, all thawed samples had significantly higher TBARS values than fresh samples. These authors explained that the addition of salt may increase the heat transfer, thereby reducing thawing time and decreasing the rate of lipid oxidation.

### 3.8. Pulsed Electric Fields

Pulsed electric fields (PEF) is a novel non-thermal technology for food preservation that involves the application of high intensity electric fields with short pulses (milliseconds or microseconds) between 0.1 and 100 kV/cm in a food matrix located between two electrodes [96]. PEF can inactivate microorganisms and inhibit several enzymes such as lipoxygenase, which is involved in the oxidative degradation of lipids [97,98]. PEF has been used to preserve liquid foods such as juices, milks, and soups, due to the higher efficiency of the electrical current transfer in liquid media [98]. However, studies on the preservation of solid foods by PEF are still scarce, especially those related to the lipid quality of fish (Table 2).

Muscle cubes of Atlantic salmon (*Salmo salar*) were exposed to PEF (1 kV/cm, 200 μs pulses) during freezing (at −18 °C) and thawing (at 10 °C) for 8 days, and TBARS levels were compared to control samples (frozen and thawed under the same conditions but not exposed to PEF) and with samples stored at 4 °C [99]. These authors found that PEF-treated frozen fish had slightly but not significantly higher TBARS values than untreated frozen samples (0.4 and ~0.36 mg MDA/kg respectively), although these values were significantly lower than those of the sample stored at 4 °C (~0.7 mg MDA/kg), reflecting a positive effect on lipid quality due to the storage at lower temperature but no significant effect of the PEF treatment. In this sense, other authors have reported an increase in lipid oxidation rates in PEF-treated meat samples, which could be due to the exposure of unsaturated fatty acids in the phospholipids of the lipid bilayer to pro-oxidant compounds as a result of the cell membrane damage induced by PEF, among other possible causes [97]. A key issue in this regard may be the intensity of the PEF treatment, to reach a high level capable of inactivating pro-oxidant enzymes in fish tissues but without causing deleterious effects on cell membranes and avoiding the promotion of lipid oxidation. In this sense, another study evaluated the effect of a low-intensity PEF treatment (500 kV/m, i.e., 5 kV/cm) on TBARS values in tilapia muscle stored at 4 °C for 6 days compared to a control without the PEF treatment, and the results showed significantly lower values in PEF-treated samples (0.27 and 0.37 mg MDA/kg for treated and non-treated samples respectively), indicating a beneficial effect of low-intensity PEF on the lipid quality of tilapia fish [100].

**Table 2:** Influence of low temperature storage combined with other preservation techniques on lipid quality indices of several fish species.

Species	Preservation Techniques	Lipid Quality Indexes	Publication Year	Main Findings	Reference
*Acipenser gueldenstaedtii* (Russian sturgeon)	Low temperature storage (−18 °C, 360 days) + vacuum packaging.	FFA: from 1.10 (fish before freezing) to 2.27 and 1.95 g/kg fish for air- and vacuum-packaged samples respectively (day 360).	2011	The use of vacuum packaging significantly reduced lipid hydrolysis of fish compared with conventional air packaging.	[78]
*Arnoglossus laterna* (scald fish)	Low temperature storage (2 °C, 12 days) + ozone cycles at days 0, 2, 5, 7, 9 and 12 (8 ppm ozone each)	FFA: from ~0.6 (day 0) to ~1.1% oleic acid (day 12) for control samples (without ozone treatment); from ~0.6 (day 0) to ~1.0% oleic acid (day 12) for treated samples. TBARS: from ~0.2 (day 0) to ~0.1 mg MDA/kg (day 12) for control samples; from ~0.2 (day 0) to ~0.4 mg MDA/kg (day 12) for treated samples.	2018	The treatment with ozone had no significant effect on inhibiting lipid oxidation in chilled fish, although TBARS values did not overpass acceptable limits of lipid quality.	[88]
*Cyprinus carpio* (common carp)	Low temperature storage (−18 °C, 180 days) + ultrasound-assisted immersion freezing (175 W, 30 kHz).	TBARS: from ~0.2 (day 0) to ~0.48 mg MDA/kg (day 180) for control (air frozen) samples; from ~0.2 (day 0) to ~0.43 mg MDA/kg (day 180) for immersion frozen samples; from ~0.2 (day 0) to ~0.37 mg MDA/kg (day 180) for ultrasonicated frozen samples.	2019	Ultrasound-assisted freezing was more efficient than air freezing (control samples) or immersion freezing to control lipid oxidation. Although TBARS levels increased over the course of the experiment in all groups, they were significantly lower at the end of the experiment in ultrasonicated samples.	[90]
*Gadus morhua* (Atlantic cod)	Low temperature storage (−20 °C, 1 week) + E-beam irradiation (0–10 kGy).	TBARS: from 0.11 mg MDA/kg for no irradiated samples (control) at the end of the freezing period to 0.17–0.18 mg MDA/kg for irradiated samples 2 to 10 kGy without significant differences among them but significantly higher than the control.	2022	TBARS values in the irradiated samples were always higher Than control fish samples, and no clear dose-effect was observed between TBARS and irradiation intensity.	[81]
*Lethrinus atkinsoni* (yellow-tailed emperor)	Low temperature storage (−18 °C, 40 days) + vacuum packaging.	FFA: from 0.62% (day 0) to 0.86% oleic acid (day 40) for control samples; from 0.62 to 0.81% oleic acid for vacuum-packaged samples. PV: from 0.92 (day 0) to 2.35 meq O_2_/kg (day 40) for control samples; from 0.93 to 2.12 meq O_2_/kg for vacuum-packaged samples. TBARS: from 0.75 (day 0) to 0.92 mg MDA/kg (day 40) for control samples; from 0.76 to 0.90 mg MDA/kg for vacuum-treated samples.	2020	Values of FFA, PV, and TBARS increased in both groups, but they were significantly lower in the vacuum-stored samples, suggesting a positive effect of vacuum on the shelf life of the fillet lipids	[79]
*Merluccius merluccius* (hake)	Low temperature storage (2 °C, 12 days) + ozone cycles at days 0, 2 (3.5 ppm ozone each), 5, 7, 9 and 12 (4.7 ppm ozone each)	FFA: from ~0.75 (day 0) to ~1.1% oleic acid (day 12) for control samples (without ozone treatment); from ~0.75 (day 0) to ~1.4% oleic acid (day 12) for treated samples. TBARS: from ~0.1 (day 0) to ~0.9 mg MDA/kg (day 12) for control samples; from ~0.1 (day 0) to ~0.4 mg MDA/kg (day 12) for treated samples.	2018	The ozone treatment significantly inhibit lipid oxidation more efficiently than using low temperature storage only according to the TBARS values.	[88]
*Oncorhynchus kisutsh* (coho salmon)	Low temperature storage (4 °C, 20 days) + HHP treatment (135–200 MPa, 30 s holding time).	FFA: from 36.5 (day 0) to 328.85 g/100 g muscle for control samples; 337.24, 333.51 and 244.33 g/100 g for pressurized samples at 135, 170 and 200 MPa respectively.	2010	No significant effect on lipid hydrolysis was observed between the control group (without pressure pre-treatment) and the tissues assayed at 135 and 170 MPa, but those subjected at the highest pressure (200 MPa) did show a delay of FFA formation compared to the control group.	[73]
*Oncorhynchus kisutsh* (coho salmon)	Low temperature storage (−18 °C, 18 months) + vacuum packaging or vacuum plus polyphenol film.	PV: from 3.81 (month 0) to 25.73 meq O_2_/kg (month 18) for control untreated samples; 22.9, 16.6 and 16.3 meq O_2_/kg (month 18) for vacuum packaged samples, vacuum packaged samples with 48 μg/dm^2^ phenolic concentration in the film, and vacuum packaged samples with 128 μg/dm^2^ phenolic concentration in the film.	2012	PV values in the control group at the end of the storage time were significantly higher than in the three groups applying vacuum, showing an inhibitory activity of primary lipid oxidation with the use of vacuum. The use of the phenol-rich film led to a not significant decrease in PV compared with using only vacuum.	[77]
*Oncorhynchus mykiss* (rainbow trout)	Low temperature storage (4 °C, 12 days) + chitosan coating/chitosan and LCS coating.	TBARS: 5.01 mg MDA/kg (Control group, no coating); 4.42 mg MDA/kg (chitosan coating); 4.48 mg MDA/kg (chitosan + LCS coating).	2015	Lipid oxidation increased in all groups at day 12 compared to day 0. However, TBARS values were significantly lower in chitosan and chitosan + LCS coating groups than in the control uncoated group.	[51]
*Oncorhynchus mykiss* (rainbow trout)	Low temperature storage (4 °C, 16 days) + *Spirulina platensis* extract or *Chlorella vulgaris* extract at 0.05 and 0.1%.	PV: from ~1 (day 0) to ~4.5 meq O_2_/kg lipids (day 16) for control samples; from ~1 to ~4 meq O_2_/kg for samples with SP and CV extracts at 0.05%; from ~1 to ~3 meq O_2_/kg for samples with SP and CV extracts at 0.1%. TBARS: from ~0.5 (day 0) to ~4.2 mg MDA/kg (day 16) for control samples; from ~0.5 to ~3.8 mg MDA/kg for samples with SP and CV extracts at 0.05%; from ~0.5 to ~2.5 mg MDA/kg for samples with SP and CV extracts at 0.1%.	2019	Extracts of SP and CV efficiently inhibited lipid oxidation when assayed at 0.1%, whereas assays at 0.05% showed PV and TBARS values closer to control samples.	[67]
*Oncorhynchus mykiss* (rainbow trout)	Low temperature storage (4 °C, 15 days) + antioxidant additives with or without alginate coating.	TBARS: from ~3 (day 0) to ~8.5 mg MDA/kg (day 15) for control (uncoated samples); from ~3 to ~7 mg MDA/kg (day 15) for alginate-coated samples; from ~3 to ~2.5–4.5 mg MDA/kg (day 15) for coated or uncoated samples with antioxidant additives.	2020	Lipid oxidation was dependant on the storage time. Alginate coating preserved the lipids more efficiently compared to uncoated samples. Antioxidant additives together with alginate coating led to the lowest TBARS values.	[53]
*Oreochromis niloticus* (tilapia)	Low temperature storage (−18 °C, 90 days) + tocopherol acetate added to fish feed (up to 200 mg/kg diet)/tocopherol acetate added post-mortem (0.1 mg/g).	TBARS: 2.8 (day 0) to 4.8 mg MDA/kg (day 90) for control samples (withouth added tocopherol); 0.9 to 2.5 mg MDA/kg for samples with 200 mg/kg from feed; 1.7 to 2.1 mg MDA/kg for samples with 200 mg/kg from feed and 0.1 mg/g added post-mortem	2007	Fish flesh enriched in tocopherol acetate is more resistant to lipid oxidation than control samples. The combination of adding tocopherol to fish feed plus adding tocopherol post-mortem is the most efficient alternative among all assayed methods to prevent lipid oxidation.	[58]
Oreochromis niloticus * (tilapia)	Low temperature storage (4 °C for 6 days) + low-intensity pulsed electric fields (5 kV/cm).	TBARS: from 0.23 (day 0) to 0.37 mg MDA/kg (day 6) for control samples (withouth PEF); from 0.23 (day 0) to 0.27 mg MDA/kg (day 6) PEF-treated samples.	2022	Low-intensity PEF significantly decreased lipid oxidation in refrigerated samples stored at 4 °C for 6 days.	[100]
*Oreochromis* sp. (tilapia)	Low temperature storage (0 °C, 77 days) + Gamma irradiation (1 and 3 kGy).	FFA: from ~9% (before irradiation) to ~21% (day 56) for control (no irradiated) samples; from ~9% to ~27% (day 56) for 1 kGy-irradiated samples; from ~9% to ~15% (day 77) for 3 kGy-irradiated samples. PV: from 50 (before irradiation) to ~80 meq O_2_/kg (day 56) for control samples; from 50 to ~130 meq O_2_/kg (day 77) for 1 kGy-irradiated samples; from 50 to ~80 meq O_2_/kg (day 77) for 3 kGy-irradiated samples. TBARS: from ~0.018 (before irradiation) to ~0.003 mg MDA/kg (day 56) for control samples; from ~0.018 to ~0.003 mg MDA/kg (day 70) for 1 kGy-irradiated samples; from ~0.018 to ~0.075 mg MDA/kg (day 70) for 3 kGy-irradiated samples.	2018	The intensity of the irradiation treatment had a significant influence on lipid oxidation indices.	[84]
*Pseudosciaena crocea* (large yellow croaker)	Low temperature storage (4 °C, 21 days) + flake ice (FI), slurry ice (SI) or ozone slurry ice (OSI) treatments.	TBARS: from ~0.4 (day 0) to ~4.2 mg MDA/kg (day 21) for FI samples; from ~0.4 (day 0) to ~3.6 mg MDA/kg (day 21) for OSI samples; from ~0.4 (day 0) to ~3.2 mg MDA/kg (day 21) for SI samples.	2023	TBARS values were gradually increased with the storage time in all groups, being this trend more pronounced in FI samples compared with SI and OSI groups, which shows a preventive effect on lipid oxidation in SI and OSI samples. However, no positive impact on lipid oxidation was shown for OSI versus SI.	[86]
*Pseudosciaena crocea* (large yellow croaker)	Repeated cycles of freezing (−20 °C for 24 h) and thawing (4 °C for 12 h) + ultrasound-assisted immersion freezing (200 W, 25 kHz).	TBARS increased 10.4-fold and 2.7-fold after 5 freeze-thaw cycles in control samples (air freezing) and ultrasound-treated samples respectively.	2023	Ultrasound-assisted immersion was an effective technique for preventing lipid oxidation after multiple freeze-thaw cycles.	[91]
*Salmo salar* (Atlantic salmon)	Low temperature storage (−18 °C, 8 days) + pulsed electric fields (1 kV/cm, pulses of 200 μs).	TBARS: 0.4, ~0.36 and ~0.7 mg MDA/kg at the end of the experiment for PEF-treated samples, control samples (frozen but without PEF treatment) and refrigerated samples (4 °C instead of −18 °C) respectively.	2020	Frozen samples showed significantly lower TBARS values than refrigerated samples, but no significant effect was found between frozen samples treated or not with PEF, reflecting a positive effect on lipid quality due to the storage at lower temperature but not due to the PEF treatment.	[99]
*Sardina pilchardus* (sardine)	Low temperature storage (−18 °C, 9 months) + HHP treatment (125–200 MPa, 0 min holding time).	PV: from 1.89 (month 0) to 1.83 meq O_2_/kg (month 9) for unpressurized control samples; 1.54 meq O_2_/kg (month 9) for treated samples at 200 MPa. TBARS: from 0.50(month 0) to 0.76 mg MDA/kg (month 9) for control samples; from 0.2–0.4 (month 0) to 0.7–0.8 mg MDA/kg (month 9) for pressurized samples.	2017	Significantly lower PV were found in frozen samples pre-treated with HHP compared to the control after 3 months of frozen storage, but a general trend could not be established for the whole duration of the experiment. Furthermore, no clear relationship was found between TBARS values and HHP-treated samples regardless the intensity of the applied pressure.	[74]
*Scomber scombrus* (Atlantic mackerel)	Low temperature storage (−10 °C, 83 days) + phenolic compounds from viticulture by-products (0.01% *w/w*) or propyl gallate.	TBARS: from ~0.3 (day 0) to ~4.5 mg MDA/kg (day 83) for control samples; from ~0.3 (day 0) to ~2–3 mg MDA/kg (day 83) for samples with phenolic extracts; from ~0.3 (day 0) to ~1.4 mg MDA/kg (day 15) for samples with propyl gallate.	2005	Phenolic extracts and propyl gallate retarded lipid oxidation in frozen fish. However, while grape phenols are soluble in water, propyl gallate is not, and therefore only the former are suitable for immersion techniques.	[55]
*Scomber scombrus* (mackerel)	Low temperature storage (−10 °C, 3 months) + HHP treatment (150–450 MPa, 0–5 min holding time).	FFA: from 1.34 (day 0) to 4.10 g/100 g lipids (3 months) for control samples; 2.79, 1.98 and 1.39 g/100 g for samples at 150, 300 and 450 MPa respectively (3 months). PV: from 1.10 to 2.97 meq O_2_/kg for control samples; 1.10, 0.85 and 1.03 meq O_2_/kg for samples at 150, 300 and 450 MPa respectively (3 months). TBARS: from 0.50 to 0.88 mg MDA/kg for control samples; 0.97, 0.81 and 0.74 mg MDA/kg for samples at 150, 300 and 450 MPa respectively (3 months).	2013	The HHP treatment had only a minor influence on the PV, and TBARS values showed no clear trend among treatment and control groups at any of the assayed conditions.Those treatments at higher pressure (450 MPa) and time (5 min) resulted in a significant inhibition of lipid hydrolysis.	[71]
*Scomber scombrus* (Atlantic mackerel)	Low temperature storage (−18 °C, 6 months) + plant extracts (from green tea, grape seeds, pomegranate rind) at 100 ppm/BHT.	PV: from ~2 (day 0) to ~7 meq O_2_/kg lipids (6 months) for BHT-added samples; from ~5–9 to ~8–11 meq O_2_/kg lipids for control and plant extract-added samples. TBARS: from ~2.5 (day 0) to ~6–7.5 mg MDA/kg (6 months) for BHT- and pomegranate rind extract-added samples; from ~2.5 to ~11–13 mg MDA/kg for samples with green tea and grape seed extracts; from ~2.5 (day 0) to ~14 mg MDA/kg for control samples (without antioxidant addition).	2018	BHT and the extract from pomegranate rind were the most efficient to inhibit lipid oxidation based on TBARS values.	[57]

FFA: free fatty acids; MDA: malondialdehyde; PV: peroxide value; TBARS: thiobarbituric acid reactive substances; CV: *Chlorella vulgaris*; HHP: High Hydrostatic Pressure; LCS: Lactoperoxidase-containing system; SP: *Spirulina platensis*; PEF: pulsed electric fields; BHT: Butylated hydroxytoluene; * We assume that “tilapia” refers to *Oreochromis niloticus,* though the authors did not specify genus and species in their work.

## 4. Conclusions

Although low temperature storage is one of the most commonly used methods to preserve fish quality, lipid oxidation is not completely inhibited even at freezing temperatures. Storage temperature and time are relevant factors regarding lipid hydrolysis and oxidation. Generally, the lower the temperature and the shorter the storage time, the better the lipid fraction is preserved. Highly unsaturated PUFA provided by fish such as EPA and DHA are of particular nutritional interest because of their widely known beneficial effects on human health. However, these PUFA are highly susceptible to oxidative degradation to primary and secondary oxidation products with undesirable effects on fish quality. The use of natural antioxidant preservatives, the application of high hydrostatic pressure, the design of novel and innovative packaging, and the use of irradiation, ozonation, ultrasound or pulsed electric fields techniques in combination with low temperature storage are alternatives that may extend the shelf life of fish and fish products. However, factors such as the concentration of antioxidants added or the intensity of the treatment are key issues in designing an effective process to maximize microbial inactivation and inhibition of oxidation-promoting enzymes while minimizing tissue damages. Many of these applications are still at the experimental stage, and considerations such as cost efficiency, energy consumption, sensory and nutritional profile preservation, among others, must be seriously addressed under a sustainable framework. Artificial intelligence applications may play an important role in the near future to help develop more efficient methods to preserve the lipid quality of fish and fish products.

## Data Availability

No new experimental data were generated in this review. Data sharing is not applicable to this article
